# Effects of Hyperbaric Oxygen Therapy on Inflammation, Oxidative/Antioxidant Balance, and Muscle Damage after Acute Exercise in Normobaric, Normoxic and Hypobaric, Hypoxic Environments: A Pilot Study

**DOI:** 10.3390/ijerph17207377

**Published:** 2020-10-10

**Authors:** Jinhee Woo, Jae-Hee Min, Yul-Hyo Lee, Hee-Tae Roh

**Affiliations:** 1 Department of Physical Education, College of Arts and Physical Education, Dong-A University, Busan 49315, Korea; sports@dau.ac.kr (J.W.); jop9508@naver.com (J.-H.M.); 2 Department of Taekwondo, Youngsan University, Yangsan-si 50510, Korea; health@ysu.ac.kr

**Keywords:** healthy young individuals, acute exercise, inflammation, oxidative stress, muscle damage, hypoxia, hyperbaric oxygen therapy

## Abstract

The purpose of this study was to investigate the effects of hyperbaric oxygen therapy (HBOT) on inflammation, the oxidative/antioxidant balance, and muscle damage after acute exercise in normobaric, normoxic (NN) and hypobaric, hypoxic (HH) environments. Eighteen healthy males were selected and randomly assigned to three groups: exercise in NN conditions (NN group, *n* = 6), HBOT treatment after exercise in NN conditions (HNN group, *n* = 6), and HBOT treatment after exercise in HH conditions (HHH group, *n* = 6). All subjects performed treadmill running for 60 min at 75–80% maximum heart rate (HRmax) exercise intensity under each condition. The HBOT treatments consisted of breathing 100% oxygen at 2.5 atmosphere absolute (ATA) for 60 min. Blood samples were collected before exercise (BE), after exercise (AE), and after HBOT (AH) to examine inflammation (fibrinogen, interleukin-6 [IL-6], and tumor necrosis factor-α (TNF-α)), the oxidative/antioxidant balance (derivatives of reactive oxygen metabolites (d-ROMs) and the biological antioxidant potential (BAP)), and muscle damage (creatine kinase (CK) and lactate dehydrogenase (LDH)). Plasma fibrinogen, serum IL-6, CK, and LDH levels were significantly increased AE compared to BE in all groups (*p* < 0.05). Plasma fibrinogen levels were significantly decreased AH compared to AE in all groups (*p* < 0.05), and the HNN group had a significantly lower AH compared to BE (*p* < 0.05). Serum IL-6 levels were significantly decreased AH compared to AE in the HNN and HHH groups (*p* < 0.05). Serum CK levels were significantly decreased AH compared to AE in the HHH group (*p* < 0.05). Serum LDH levels were significantly decreased AH compared to AE in the HNN and HHH groups (*p* < 0.05), and the NN and HNN groups had significantly higher AH serum LDH levels compared to BE (*p* < 0.05). These results suggest that acute exercise in both the NN and HH environments could induce temporary inflammatory responses and muscle damage, whereas HBOT treatment may be effective in alleviating exercise-induced inflammatory responses and muscle damage.

## 1. Introduction

Regular exercise training is not only effective in preventing and relieving the symptoms of metabolic diseases, such as hypertension, hyperlipidemia, and diabetes mellitus, and cardiovascular disease by reducing the levels of chronic inflammation and oxidative stress (OS) within the body, but it can also induce numerous other health benefits by increasing skeletal muscle mass [1,2]. In contrast, acute exercise, which stresses body tissues, can cause excessive OS accompanied by muscle damage [3,4]. The body exhibits decreased muscular function and/or inflammatory response due to excessive muscle fatigue without proper recovery from the damage, and this can lead to exercise impairment as well as a reduction in the ability to perform exercise [3,5]. Previous studies suggested that inflammatory cytokines, such as interleukin-6 (IL-6) and tumor necrosis factor-α (TNF-α), and fibrinogen could be blood biomarkers for the inflammatory response induced by exercise [6]. Serum creatine kinase (CK) and lactate dehydrogenase (LDH) levels are typical indices that reflect exercise-induced muscle damage [7]. In addition, exercising in hypoxic conditions can put additional stress on the body compared to exercising in normoxia [8]. Hypoxia was reported to increase the generation of reactive oxygen species, have negative impacts on antioxidant defenses, and cause severe OS and inflammation through TNF-α release and nuclear factor-κB (NF-κB) activation [9,10].

Strategies, such as consuming food (nutrients) with antioxidant and anti-inflammatory effects [11,12], low-level laser therapy [13], and cold water immersion [14] treatment, have been suggested to reduce the inflammatory response and excessive OS generation from acute exercise, and help exercise-induced damaged muscles recover faster. The strategies remain controversial, but some of them were proven to be effective. Recently, studies reported that hyperbaric oxygen therapy (HBOT) could be effective in early recovery from exercise-related muscular injury due to its beneficial effect on cell recovery and tissue repair [15,16]. HBOT is a treatment procedure where the patient is intermittently given 100% pure oxygen under pressure higher than atmospheric pressure (1 atmosphere absolute [ATA] = 101 kPa) in a hyperbaric chamber. The typical range of pressure is 2–2.8 ATA and it is effective at 1.4 ATA or greater pressure [15]. HBOT is a treatment that can be applied to various conditions, including carbon monoxide poisoning, compromised skin graft and flaps, crushing injuries, necrotizing soft tissue infections, and non-healing ulcers with arterial inefficiencies. Moghadam et al. [15] suggested this as a possible treatment for sports musculoskeletal injuries as it can enhance oxygen delivery, reduce edema and pathologic inflammation, and mitigate ischemia/reperfusion injury. Chen et al. [16] applied HBOT treatment to 41 athletes with exercise-related muscle damage and reported that muscle damage indices like CK, glutamic oxaloacetate transaminase (GOT), and myoglobin were significantly decreased.

However, previous studies [17,18] reported that HBOT treatment in the recovery phase after exercise had no significant effect in relieving exercise-induced muscle soreness and damage. Despite the experimental evidence [19] showing that HBOT treatment could mitigate OS in human endothelial cells, only a few studies have verified the effect of HBOT treatment on exercise-induced inflammation and OS in the recovery phase after exercise. Moreover, the effect of HBOT treatment after exercising in hypoxic conditions which can induce higher level of OS and more inflammatory response was not reported. The purpose of this study was to verify the levels of inflammation, OS, and muscle damage according to normobaric, normoxic and hypobaric, hypoxic exercise environments, and investigate the beneficial effect of HBOT treatment in relieving inflammation, OS, and muscle damage in the recovery phase after acute exercise.

## 2. Methods

### 2.1. Subjects

The prospective number of subjects was calculated to be 18 subjects for an effect size of 0.40, an α value of 0.05, and a desired statistical power (1-β) of 0.80. We posted a recruitment notice at Dong-A University for three weeks from 1 December 2018, and recruited 18 healthy males with normal body composition (body mass index (BMI) under 30 kg/m^2^) with no musculoskeletal disorders and no surgical experience related to that in the past one year. The 18 selected subjects were randomly assigned to three groups of six people each: one exercised in normobaric, normoxic (NN) conditions (NN group), one had HBOT treatment after exercising in NN conditions (HNN group), and the last one had HBOT treatment after exercising in hypobaric, hypoxic (HH) conditions (HHH group). Approval for this study was obtained from the Ethics Committee of Dong-A University (ID: 2-1040709-AB-N-01-201810-HR-032-04). All subjects were fully informed of the study procedures and signed an informed consent form indicating that they understood the study procedures and the risks and benefits of participation. The characteristics of the subjects at baseline are shown in Table 1. There were no significant differences between the groups.

### 2.2. Anthropometric Measures and VO_2_max Test

Anthropometric measurements, which were taken one week before beginning the main exercise test (NN and HH conditions), included height, body composition, and maximal oxygen uptake (VO_2_max). Height and body composition were measured using a multi-frequency bioelectrical impedance analyzer (Accuniq BC720; SELVAS Healthcare, Daejeon, Korea). VO_2_max was measured on a treadmill (T150; Cosmed, Rome, Italy) according to the Bruce protocol [20], using a gas analyzer (Quark CPET; Cosmed, Rome, Italy) based on the breath-by-breath method.

### 2.3. Exercise Environment Setting and HBOT Procedure Protocol

**Figure 1:**
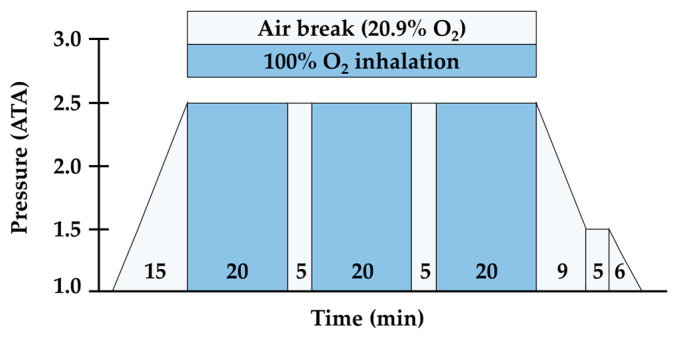
Hyperbaric oxygen therapy (HBOT) treatment protocol.

In the exercise environment setting and for HBOT treatment, a multi-pressure chamber (Interocean I.O medical, Busan, Korea) was used that could reflect the atmospheric pressure and create conditions of hypobaric/hypoxia and hyperbaric/hyperoxia. More specifically, the subjects in each group (NN, HNN, and HHH) ran on a treadmill for 60 min at 75–80% HRmax intensity in NN conditions (760 Torr, approximately 20.9% oxygen) for those at sea level and HH conditions (526 Torr, approx. 14.5% oxygen) for those at an altitude of 3000 m. Each treadmill run was initiated with the Bruce protocol [20] while wearing a Bluetooth heart rate sensor (H10; Polar Electro Oy, Kempele, Finland). When the subject’s heart rate was monitored and the target heart rate reached, the treadmill slope was fixed at 0%, and the treadmill speed was adjusted to control the exercise intensity. For HBOT treatment of the HNN and HHH groups, the protocol of inhaling 100% oxygen at an increased air pressure of 2.5 ATA was used according to a previous study by Shimoda et al. [21]. The subjects were compressed to 2.5 ATA over 15 min, after which they breathed 100% oxygen for 60 min delivered through a tight-fitting mask covering the nose and mouth for three 20-min periods with 5-min breaks in ambient air (20.9% oxygen). Two 5-min air breaks in ambient air were carried out in 2.5 ATA. At the end of the 60-min period, 15 min was allowed for the participant to decompress to atmospheric pressure. The treatment method for this study requires a 5-min air break for every 20-min oxygen inhalation to avoid oxygen toxicity. The treatment was applied for 105 min including 45 min of air breaks (Figure 1).

### 2.4. Blood Collection and Analyses

Using a 22-gauge needle, 10 mL of blood was collected from the antecubital vein of each subject before exercise (BE), after exercise (AE), and after HBOT (AH) into a serum separator tube, an ethylenediamine tetraacetate-containing tube, and a sodium citrate-containing tube (all tubes were purchased from Becton Dickinson, Franklin Lakes, NJ, USA). The collected blood samples were centrifuged for 15 min at 3000 rpm and were then stored at −80 °C until analysis. The analysis of plasma fibrinogen levels was performed using a chronometric assay (Fibriprest, Diagnostica Stago, Asnieres, France). Serum IL-6 and TNF-α levels were measured using a human IL-6 Duoset enzyme-linked immunosorbent assay (ELISA) kit (DY206; R&D Systems, Minneapolis, MN, USA) and a human TNF-alpha Quantikine HS ELISA kit (HSTA00E; R&D Systems, Minneapolis, MN, USA), respectively. A microplate reader (Sunrise^TM^; TECAN Austria GMBH, Grödig/Salzburg, Austria) was used to measure the absorbance at 450 nm for quantification. The analyses of serum derivatives of reactive oxygen metabolites (d-ROMs) and the biological antioxidant potential (BAP) levels were measured using a commercial assay kit (Diacron SRL, Proma, Italy) as described by Hussein et al. [22]. Serum CK and LDH levels were measured at 680 nm absorbance using a standard microwell plate reader (Molecular Devices, Orleans, CA, USA).

### 2.5. Statistical Analyses

Statistical analyses were performed with SPSS version 25.0 for Windows (SPSS Inc., Chicago, IL, USA). The data are presented as the mean ± standard deviation. A two-way repeated analysis of variance (ANOVA) was performed to identify the differences in normally distributed data. Tests of normality for all measured values were conducted using the one-sample Kolmogorov–Smirnov test. When significant group by time interactions occurred, the simple main effects were assessed using a one-way ANOVA. The Tukey post hoc test was used as a conservative locator of significant differences. Statistical significance was set at *p* < 0.05.

## 3. Results

### 3.1. Changes in Variables Related to Inflammation

**Figure 2:**
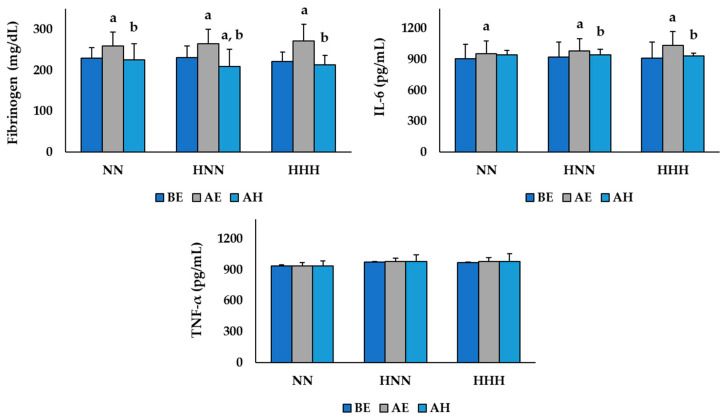
Changes in inflammatory markers according to HBOT after exercise in NN and HH environments. Values are mean ± standard deviation; NN: exercised in normobaric, normoxic conditions; HNN: HBOT treatment after exercising in normobaric, normoxic conditions; HHH: HBOT treatment after exercising in in hypobaric, hypoxic conditions; BE: before exercise; AE: after exercise; AH: after HBOT; IL-6: interleukin-6; TNF-α: tumor necrosis factor-α; ^a^ Significant difference with BE (*p* < 0.05); ^b^ Significant difference with AE (*p* < 0.05).

The changes in inflammatory markers according to HBOT after exercise in the NN and HH environments are shown in Figure 2. The two-way repeated ANOVA demonstrated a significant difference in the group by time interaction for plasma fibrinogen (*F*  =  3.356, *p*  =  0.022) and serum IL-6 (*F*  =  13.059, *p*  <  0.001) levels. Plasma fibrinogen levels were significantly increased AE compared to BE in all groups, and all groups were significantly decreased AH (*p* < 0.05). In addition, the HNN group had significantly lower AH plasma fibrinogen levels compared to BE (*p* < 0.05). Serum IL-6 levels were significantly increased AE compared to BE in all groups, and the levels in the HNN and HHH groups were significantly decreased AH (*p* < 0.05). In contrast, the serum TNF-α (*F* = 1.131, *p* = 0.361) levels were not significantly different between any groups or time points.

### 3.2. Changes in Variables Related to Oxidative/Antioxidant Balance

**Figure 3:**
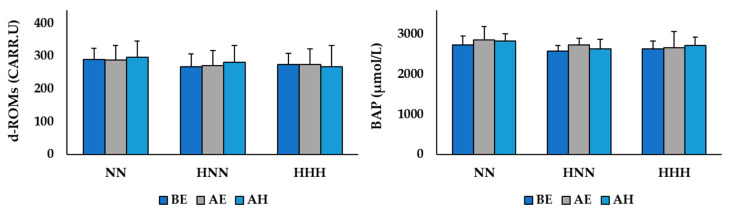
Changes in oxidative/antioxidant balance markers according to HBOT after exercise in NN and HH environments. Values are mean ± standard deviation; NN: exercised in normobaric, normoxic conditions; HNN: HBOT treatment after exercising in normobaric, normoxic conditions; HHH: HBOT treatment after exercising in in hypobaric, hypoxic conditions; BE: before exercise; AE: after exercise; AH: after HBOT d-ROMs: derivatives of reactive oxygen metabolites; BAP: biological antioxidant potential.

The changes in oxidative/antioxidant balance markers according to HBOT after exercise in the NN and HH environments are shown in Figure 3. The two-way repeated ANOVA demonstrated no significant difference in the group by time interaction for serum d-ROMs (*F*  =  0.512, *p*  =  0.728) and BAP (*F*  =  0.657, *p*  =  0.626) levels.

### 3.3. Changes in Variables Related to Muscle Damage

**Figure 4:**
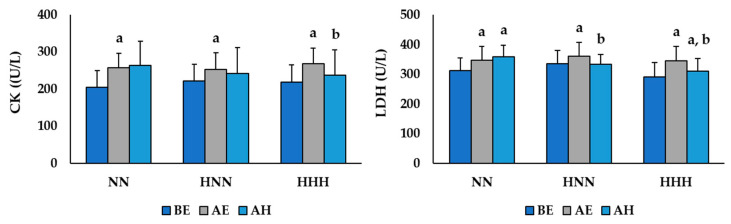
Changes muscle damage markers according to HBOT after exercise in NN and HH environments. Values are mean ± standard deviation; NN: exercised in normobaric, normoxic conditions; HNN: HBOT treatment after exercising in normobaric, normoxic conditions; HHH: HBOT treatment after exercising in in hypobaric, hypoxic conditions; BE: before exercise; AE: after exercise; AH: after HBOT; CK: creatine kinase; LDH: lactate dehydrogenase; ^a^ Significant difference with BE (*p* < 0.05); ^b^ Significant difference with AE (*p* < 0.05).

The changes in muscle damage markers according to HBOT after exercise in the NN and HH environments are shown in Figure 4. The two-way repeated ANOVA demonstrated a significant difference in the group by time interaction for serum CK (*F*  =  6.880, *p*  <  0.001) and LDH (*F*  =  10.362, *p*  <  0.001) levels. Serum CK levels were significantly increased AE compared to BE in all groups, and the level in the HHH group was significantly decreased AH (*p* < 0.05). Serum LDH levels were significantly increased AE compared to BE in all groups, and the levels in the HNN and HHH groups were significantly decreased AH (*p* < 0.05). In addition, the NN and HNN groups had significantly higher serum LDH levels AH compared to BE (*p* < 0.05).

## 4. Discussion

Inflammation is a reaction that causes fever and pain in the body in response to foreign antigens and tissue damage, such as in skeletal muscles. Even though it is a necessary defensive reaction that helps damaged tissue to recover, it can also cause other diseases like cardiovascular disease [23] and is closely related to musculoskeletal damage and muscle fatigue [24,25]. In some previous studies [6,26], the levels of fibrinogen, IL-6, and TNF-α in the blood have been reported to be indices reflecting the inflammatory response induced by acute exercise. This study analyzed the levels of plasma fibrinogen, serum IL-6, and TNF-α to verify changes in the inflammatory response depending upon the exercise performed in NN and HH environments and investigated the effect of HBOT on recovery. The results of this study showed that the levels of plasma fibrinogen and serum IL-6 significantly increased after exercise in all groups, but there was no significant difference between the exercise environments (NN vs. HH). These results are consistent with previous studies [6,27] that demonstrated that acute exercise significantly increased the levels of fibrinogen and IL-6 in the blood by causing an inflammatory response, and suggest that exercising in an HH environment, which corresponds to conditions at an altitude of about 3000 m, did not lead to additional inflammatory responses compared to exercising in an NN environment. Liakos et al. [6] reported that the levels of fibrinogen and IL-6 were significantly increased after maximal treadmill exercise testing using the Bruce protocol, and in Stelzer et al.’s study [27], the levels of fibrinogen and IL-6 were analyzed to verify changes in the inflammatory response with ultra-endurance exercise. The results showed that the fibrinogen and IL-6 levels were significantly increased after the race. Furthermore, Kasai et al. [26] conducted a repeated sprint exercise with 10 male athletes in hypoxic (fraction of inspired oxygen (F_i_O_2_ = 14.5%, equivalent to a simulated altitude of 3000 m) and normoxic (F_i_O_2_ = 20.9%) conditions. Although the levels of IL-6 increased significantly after exercise in both conditions, there was no significant difference in levels between the two environmental conditions, which supports the results of this study. Additionally, the plasma fibrinogen levels were significantly lower at the BE point than at the AH point in the HNN group in this study. Additionally, the level of serum IL-6 decreased significantly at the AH point compared to the AE point in both the HNN and HHH groups, but there was no significant decrease in the NN group. This implies that HBOT treatment after exercise can be effective in relieving exercise-induced inflammation, supporting the findings of previous studies [28] that HBOT treatment could help athletics recover while training due to its effect to relieve inflammation.

Oxygen is a crucial factor in generating energy (adenosine triphosphate), which is required by the body for use in various metabolic processes when the body is resting and also when it is exercising and recovering. However, the incomplete reduction of oxygen during metabolic processes produces reactive oxygen species (ROS) that can be toxic to the body. Furthermore, the levels of OS in the body increase due to the accelerated production of ROS during highly intensive exercise, which requires a greater oxygen supply [3]. This study analyzed the levels of serum d-ROMs and BAP to verify the oxidative/antioxidant balance after exercising in NN and HH environments and investigated the effect of HBOT treatment on recovery. The levels of serum d-ROMs and BAP were used as indices of exercise-induced oxidative stress in a number of previous studies [29,30,31] as they reflect the level of OS and antioxidant capacity, respectively. Specifically, Sugita et al. [29] and Martarelli et al. [30] reported that the levels of d-ROMs and BAP increased significantly after incremental cycle exercise and mountain bike exercise in separate studies, and the levels of d-ROMs and BAP increased significantly, and the levels were also increased after cycling in 75% VO_2_max intensity in Aoki et al.’s study [31]. However, in this study, no statistically significant difference was found in either the d-ROMs or BAP levels. It is considered that high antioxidant activity and high levels of maximal aerobic power (VO_2_max) of the participants in this study played major roles in the study results. That is, OS production in the body caused from an imbalance between pro-oxidants and antioxidants, such as excessive ROS production and/or compromised intrinsic antioxidant defense [32], may have contributed to the higher resting level serum BAP of the participants in this study at 2647.61 ± 245.45 μmol/L than the resting level BAP (from 1938.5 to 2347.3 μmol/L) in previous studies [29,30,31], which proved that the d-ROMs and BAP levels increased after acute exercise. Additionally, the VO_2_max level of the participants in this study was 48.20 ± 2.19 mL/kg/min which is above the 60th percentile for adult men in their 20s [33]. Bachi et al. [34] suggested that the level of OS after exercise could be lower in people with higher VO_2_max and total antioxidant activity. However, considering that high-intensity acute exercise can be one of the main causes of increased OS in the body, it seems necessary to verify this in additional studies by analyzing serum ROS levels and/or antioxidant enzymes such as superoxide dismutase (SOD) and catalase, not only d-ROMs and BAP levels.

As exercise-induced muscle damage is caused by acute or long-term damage to muscle cells and tissues, it is possible to indirectly estimate the level of damage from the levels of enzymes like CK and LDH that are released into the blood from muscle tissues during exercise [7,15]. Serum muscle enzyme levels increase not only in the body of athletes who exercise intensively but also in ordinary people after exercise. The increased inflammatory response and repetitive muscle damage by exercising can cause injury, negatively impact the length of rehabilitation after an injury, and reduce the ability to exercise [7,15,16]. In this study, we analyzed the levels of serum CK and LDH to verify the changes in muscle damage after exercise in the NN and HH environments, and establish the recovery effect of HBOT treatment.

The results of this study showed that the serum CK and LDH levels were significantly increased after exercising in all groups, and there was no significant difference between the exercise environments (NN vs. HH). This result implies that treadmill running at an intensity of 75–80% HRmax can cause muscle damage, and the HH environment, which was the same as that at an altitude of about 3000 m, did not add additional stress to the exercise-induced muscle damage. Kasai et al. [26] reported that serum myoglobin levels, the index of muscle damage in blood, increased significantly, irrespective of hypoxic or normoxic conditions. Sumi et al. [35] also suggested that endurance exercise in moderately hypoxic conditions (F_i_O_2_ = 14.5%) did not facilitate more exercise-induced muscle damage response or cause in endurance athletes than in normoxic conditions (F_i_O_2_ = 20.9%). In addition, the serum CK levels were significantly decreased in the HHH group, and the serum LDH levels were significantly decreased in the HNN and HHH groups at the AE point compared to the AH point. These results suggested that HBOT treatment in the recovery phase after exercise was effective in mitigating exercise-induced muscle damage. The study by Cervaens et al. [36], which conducted HBOT treatment of rats with muscle injuries, reported that the serum CK levels were significantly decreased and that HBOT was effective for muscle injury recovery. Although some previous studies [17,37,38] conducted in humans reported that HBOT treatment after exercise did not affect muscle damage (injury) or muscular pain relief, additional studies with larger numbers of subjects should be conducted to verify the effect of HBOT.

The limitations of this study were that (1) the number of subjects was small as this is a pilot study, and (2) serum CK and LDH, the indices of muscle damage, tend to continuously increase up to 72 h after exercise. Therefore, further verification in additional studies is necessary, taking into account the half-life of CK and LDH.

## 5. Conclusions

In conclusion, acute exercise in the NN and HH environments caused a temporary inflammatory response and muscle damage, but there was no difference between the environmental conditions. In contrast, HBOT treatment in the recovery phase had a positive impact on relieving the inflammatory response and muscle damage after exercise.

## Figures and Tables

**Table 1:** Characteristics of the subjects at baseline.

Variable/Group	NN (*n* = 6)	HNN (*n* = 6)	HHH (*n* = 6)	*p* Value ^&^
Age (years)	23.67 ± 3.44	21.67 ± 2.34	23.00 ± 2.76	0.490
Height (cm)	174.33 ± 2.89	173.87 ± 4.30	176.92 ± 5.55	0.450
Weight (kg)	75.63 ± 4.80	72.95 ± 5.23	74.43 ± 6.03	0.694
BMI (kg/m^2^)	24.88 ± 1.53	24.12 ± 1.29	23.75 ± 0.68	0.290
Body fat (%)	22.28 ± 7.35	21.20 ± 5.23	20.97 ± 3.08	0.907
HRrest (beats/min)	73.33 ± 8.82	70.33 ± 10.75	66.00 ± 6.87	0.385
HRmax (beats/min)	196.33 ± 3.44	198.33 ± 2.34	197.00 ± 2.76	0.490
VO_2_max (mL/kg/min)	47.17 ± 2.30	49.15 ± 2.82	48.29 ± 0.87	0.308
Fibrinogen (mg/dL)	230.00 ± 24.92	231.67 ± 33.68	221.83 ± 39.63	0.862
IL-6 (pg/mL)	909.17 ± 136.62	924.47 ± 125.85	912.83 ± 48.01	0.969
TNF-α (pg/mL)	938.80 ± 8.52	975.46 ± 34.59	971.19 ± 51.42	0.192
d-ROMs (CARR.U)	289.67 ± 34.07	267.83 ± 44.21	274.67 ± 50.54	0.680
BAP (μmol/L)	2726.67 ± 220.81	2583.67 ± 329.77	2632.50 ± 186.14	0.619
CK (U/L)	205.85 ± 44.02	221.58 ± 38.27	218.50 ± 64.36	0.849
LDH (U/L)	312.83 ± 42.98	335.67 ± 45.91	291.50 ± 39.67	0.237

Values are mean ± standard deviation. NN: exercised in normobaric, normoxic conditions; HNN: HBOT treatment after exercising in normobaric, normoxic conditions; HHH: HBOT treatment after exercising in in hypobaric, hypoxic conditions; HRrest: heart rate at rest, HRmax: heart rate at peak exercise; VO_2_max: maximal oxygen uptake; IL-6: interleukin-6; TNF-α: tumor necrosis factor-α; d-ROMs: derivatives of reactive oxygen metabolites; BAP: biological antioxidant potential; CK: creatine kinase; LDH: lactate dehydrogenase; ^&^ Determined using the one-way ANOVA.
